# Psychological state and its correlates of local college students in Wuhan during COVID‐19 pandemic

**DOI:** 10.1002/pits.22699

**Published:** 2022-04-25

**Authors:** Fenghuixue Liu, Luojia Dai, Yuyang Cai, Xin Chen, Jiaqing Li, Lili Shi

**Affiliations:** ^1^ School of Public Health, Shanghai Jiao Tong University School of Medicine Shanghai China; ^2^ Xinhua Hospital, Shanghai Jiao Tong University School of Medicine Shanghai China

**Keywords:** administrators, counselors, psychology, school psychologists, schools, teachers

## Abstract

In 2020, the lockdown of Wuhan due to the outbreak of COVID‐19 impacted various aspects of local college students' life and may further negatively affect their psychological state. This study was conducted among 652 Wuhan local college students during the quarantine of this city. We assessed their psychological state using Depression‐Anxiety‐Stress Scale 21 and evaluated their living condition including diet, schedule, recreational activities, social contact, academic life, and attention paid to pandemic news. Results showed that 16.87% of the students reported stress, 28.68% with anxiety, and 35.12% had depression. According to multivariate logistic regression analysis, having a medical background was associated with higher stress levels; students who had an irregular diet and schedule were more likely to develop stress, anxiety, and depression; students with their academic life affected had a higher prevalence of anxiety and depression. By studying local students in the hardest‐hit area during the pandemic, our findings can provide references for the improvement of college students' mental health in the long term.

## Highlights

- Wuhan local college students developed different severity of stress, anxiety, and depression during the quarantine.
- Students with a medical background showed higher stress levels.
- Students who had an irregular diet and schedule were more likely to develop stress, anxiety, and depression.
- Students with their academic life affected had a higher prevalence of anxiety and depression.

## 1 INTRODUCTION

Coronavirus 2019 (COVID‐19) outbroke in December 2019 and spread rapidly across the world (WHO, 2020). On January 23, 2020, the Wuhan government announced the traffic administration in the city and the closeup of passages in and out of it (hereinafter referred to as “the quarantine”). Due to the suddenness and urgency of the pandemic, many local people went through the quarantine with various aspects of their lives impacted. Although the first round of outbreak was contained and Wuhan unlocked on April 8, 2020, the pandemic continued and China has stepped into a new phase of regular epidemic prevention and control.

The outbreak itself is a source of stress for the public because of the increasing number of patients and affected countries around the world. Further executive orders trying to contain the outbreak (e.g., closed management and traveling bans of the whole or part of the affected areas) may also generate public anxiety (Bao et al., 2020). For example, Lei et al. (2020) found that the prevalence of anxiety and depression in people affected by quarantine during COVID‐19 was significantly higher than those of people in unaffected areas, which proved the negative psychological impacts of the pandemic. In addition, people who lived in the hardest‐hit areas of the pandemic might face more severe psychological problems (N. Liu, Zhang, et al., 2020; Qiu et al., 2020) which calls for more attention to mental health of people quarantined in the hardest‐hit areas.

Previous research found that student status was significantly associated with a greater psychological impact of the outbreak (C. Wang, Pan, Tan, Xu, et al., 2020). Moreover, one study on the age of onset of mental disorders showed that college students, who are going through the transition from adolescents to adults (Auerbach et al., 2018), are at a peak period of getting emotional disorders (de Girolamo et al., 2012), indicating they are in more vulnerable mental status compared to adults. (X. Wang, Hegde, et al., 2020). In the early stages of the pandemic, studies on Chinese college students found scores of stress, anxiety, and depression were higher than the normal mode (Bian & Zhao, 2020; Chen & Hao, 2020; Gao et al., 2020). Moreover, as colleges and universities were closed and online learning had been done on such a large scale for the first time in China due to the pandemic, local college students under quarantine in Wuhan not only need to overcome the inconvenience of living in isolation but also had to deal with the change of academic schedule and adapt to new ways of learning. All these changes may elevate the risk of developing mental disorders.

Therefore, studying local college students in Wuhan during COVID‐19 has a profound significance for psychological research. In this study, the psychological state of local college students in Wuhan and its correlates like diet, schedule, recreational activities, social contact, and academic life were explored, so as to provide references for improving the mental health of college students during pandemic through the analysis of this special group.

## 2 METHODS

### 2.1 Survey respondents

From March 7, 2020, to March 15, 2020, online questionnaires were distributed through a Chinese online research panel, Tencent Survey (https://wj.qq.com/), to survey local college students in Wuhan, using snowball and convenience sampling. A total of 708 questionnaires were distributed and 652 of them were valid, with an effective rate of 92.09%.

### 2.2 Research tools

#### 2.2.1 General condition questionnaire

The general condition questionnaire investigated college students' general information such as gender, grade, school location, medical background, and health condition of oneself, family members, relatives, and friends. Moreover, two items “Where is your hometown?” and “Where have you been during the pandemic?” were to measure the validity of students. Those who chose “Wuhan” for both items were identified as valid respondents.

#### 2.2.2 Living condition questionnaire

The living condition questionnaire asked about college students' living information such as diet, schedule, recreational activities, social contact, academic life, and attention paid to pandemic news. Some of these were quantified and classified.

#### 2.2.3 Depression‐Anxiety‐Stress Scale 21 (DASS‐21)

DASS was first proposed by Lovibond and Lovibond (1995) and it has been proven to have a high degree of internal consistency and reliability which was later reduced to 21 questions by Antony et al. (1998). In this study, the reduced version was used to quantify the negative emotion of the respondents and to evaluate their prevalence and level of depression, anxiety, and stress.

### 2.3 Statistical methods

All data were analyzed using SPSS 24.0 (IBM). Number, composition ratio, and mean value (with standard deviation) were used as descriptive indicators. For categorical data, the *χ* ^2^ test was performed to examine the differences in the prevalence of depression, anxiety, and stress between different demographic factors and living conditions. Adjusted odds ratios (aORs) with 95% confidence intervals (CIs) were calculated to explore the influencing factors of the prevalence of three symptoms by multivariate logistic regression analysis. A *p* < .05 was considered statistically significant.

## 3 RESULTS

### 3.1 General information

Of the 652 respondents, 47.09% were males; 4.75% were in senior and above while the grades of students before senior year were evenly distributed; 34.05% had a medical background, among which 20.27% majored in medicine, 83.33% of them had families engaged in medical‐related career; 99.69% and 97.55% self‐reported good physical health of themselves and their family, while only 61.35% reported their relatives and friends not infected with COVID‐19 (Table 1).

**Table 1:** General information

		Male		Female		Total	
		*N*	%	*N*	%	*N*	%
Grade	Preparatory and freshman	95	30.94	128	37.10	223	34.20
	Sophomore	84	27.36	107	31.01	191	29.29
	Junior	110	35.83	97	28.12	207	31.75
	Senior and above	18	5.86	13	3.77	31	4.75
School location	Wuhan, Hubei province	198	64.50	226	65.51	424	65.03
	Other regions	109	35.50	119	34.49	228	34.97
Medical background	Yes	99	32.25	123	35.65	222	34.05
	No	208	67.75	222	64.35	430	65.95
Health condition of oneself	Healthy	305	99.35	345	100.00	650	99.69
	Ill	2	0.65	0	0.00	2	0.31
Health condition of family	Health	297	96.74	339	98.26	636	97.55
	Ill	10	3.26	6	1.74	16	2.45
Health condition of relatives and friends	Healthy	202	65.80	198	57.39	400	61.35
	Ill	105	34.20	147	42.61	252	38.65
Total		307	47.09	345	52.91	652	100.00

### 3.2 Psychological state of local college students in Wuhan

According to the DASS‐21 scoring standard, there were 41 (6.29%), 38 (5.83%), 25 (3.83%), and 6 (0.92%) students with mild, moderate, severe, and very severe stress. The numbers of students with mild, moderate, severe, and very severe anxiety were 43 (6.60%), 97 (14.88%), 21 (3.22%), and 26 (3.99%), respectively. As for depression, 232 students (35.12%) were depressed, including 95 (14.57%) with mild depression, 97 (14.88%) with moderate depression, 18 (2.76%) with severe depression, and 19 (2.91%) with very severe depression (Table 2).

**Table 2:** Basic conditions of Depression‐Anxiety‐Stress Scale 21

	Stress		Anxiety		Depression	
	Score	*N*(%)	Score	*N*(%)	Score	*N*(%)
Normal	0–14	542 (83.13)	0–7	465 (71.32)	0–9	423 (64.88)
Mild	15–18	41 (6.29)	8–9	43 (6.60)	10–13	95 (14.57)
Moderate	19–25	38 (5.83)	10–14	97 (14.88)	14–20	97 (14.88)
Severe	26–33	25 (3.83)	15–19	21 (3.22)	21–27	18 (2.76)
Extremely severe	34+	6 (0.92)	20+	26 (3.99)	28+	19 (2.91)

### 3.3 Analysis of factors influencing the psychological state of local college students in Wuhan

#### 3.3.1 Description of the living condition factors

There were 16.9% of the local college students with irregular diets, 17.64% of the students had difficulty storing enough food in 1 week. 29.6% of the students have an irregular schedule. The proportion of students with low satisfaction with social contact and recreational activities was 37.4% and 53.4%. 81.3% of the local college students were delayed in academic life.

#### 3.3.2 Factors correlated with stress

In the unadjusted model, it is found that the risk of stress was significantly correlated with having a medical background, relatives or friends being ill, an irregular diet or schedule, academic life being delayed, and lower satisfaction with social contact and recreational activities (*p* < .05). However, after adjusting for other factors, students with a medical background (aOR = 1.674, 95% CI = 1.066–2.631) were more likely to develop stress. Besides, compared to having an irregular diet and schedule, a regular diet (aOR = 0.409, 95% CI = 0.216–0.775) and a regular schedule (aOR = 0.402, 95% CI = 0.225–0.717) were associated with lower risk of stress (Table 3).

**Table 3:** Correlates of symptoms of stress, anxiety, and depression

		Stress				Anxiety				Depression			
		cOR (95% CI)	*p* Value	aOR (95% CI)^a^	*p* Value	cOR (95% CI)	*p* Value	aOR (95% CI)^a^	*p* Value	cOR (95% CI)	*p* Value	aOR (95% CI)^a^	*p* Value
Health condition of relatives and friends													
	Healthy	0.624 (0.413–0.943)	.025	0.743 (0.477–1.158)	.190	0.810 (0.573–1.145)	.232	0.915 (0.630–1.330)	.642	0.744 (0.536–1.033)	0.078	0.821 (0.576–1.171)	.277
	Ill												
Medical background													
	Yes	1.565 (1.030–2.378)	.036	1.674 (1.066–2.631)	.025	1.192 (0.837–1.700)	.331	1.263 (0.862–1.850)	.231	1.196 (0.854–1.676)	.297	1.233 (0.857–1.773)	.259
	No												
Diet													
	Irregular												
	Regular	0.325 (0.196–0.540)	.000	0.446 (0.237–0.838)	.012	0.238 (0.150–0.376)	.000	0.369 (0.211–0.643)	.000	0.238 (0.152–0.375)	.000	0.402 (0.233–0.692)	.001
	Medium	0.298 (0.179–0.498)	.000	0.409 (0.216–0.775)	.006	0.355 (0.220–0.574)	.000	0.426 (0.246–0.737)	.002	0.376 (0.234–0.605)	.000	0.487 (0.284–0.836)	.009
Schedule													
	Irregular												
	Regular	0.273 (0.164–0.453)	.000	0.507 (0.269–0.955)	.350	0.292 (0.188–0.453)	.000	0.467 (0.273–0.801)	.006	0.263 (0.173–0.400)	.000	0.401 (0.241–0.666)	.000
	Medium	0.319 (0.184–0.554)	.000	0.402 (0.225–0.717)	.002	0.438 (0.291–0.659)	.000	0.599 (0.374–0.958)	.033	0.404 (0.272–0.600)	.000	0.521 (0.333–0.817)	.005
Satisfaction with social contact													
	High												
	Low	1.868 (1.235–2.824)	.003	1.468 (0.916–2.352)	.111	1.816 (1.285–2.567)	.001	1.375 (0.927–2.041)	.114	1.589 (1.143–2.210)	.006	1.140 (0.780–1.666)	.498
Satisfaction with recreational activities													
	High												
	Low	1.742 (1.138–2.665)	.011	1.136 (0.696–1.854)	.610	1.862 (1.312–2.642)	.000	1.284 (0.859–1.920)	.224	1.924 (1.383–2.677)	.000	1.441 (0.986–2.107)	.059
Academic life													
	Delayed	1.706 (0.937–3.108)	.080	1.779 (0.943–3.356)	.075	1.929 (1.182–3.147)	.009	2.045 (1.210–3.454)	.007	1.844 (1.178–2.886)	.007	1.964 (1.211–3.185)	.006
	Not delayed												
Attention paid to pandemic news													
	Low												
	High	0.957 (0.382–2.397)	.925	0.995 (0.371–2.666)	.992	0.924 (0.425–2.007)	.841	0.942 (0.405–2.190)	.890	1.422 (0.64–3.151)	.386	1.518 (0.642–3.586)	
	Medium	0.337 (0.107–1.059)	.063	0.358 (0.107–1.197)	.095	0.483 (0.197–1.183)	.111	0.523 (0.200–1.370)	.187	0.997 (0.413–2.406)	.995	1.165 (0.452–3.000)	

Abbreviations: aOR, adjusted odds ratio; CI, confidence interval; cOR, crude odds ratio.

^a^ Model adjusted for medical background, diet, schedule, academic life, satisfaction with social contact, and other variates.

#### 3.3.3 Factors correlated with anxiety

As shown in Table 3, the risk of anxiety in college students was significantly correlated with having an irregular diet or schedule, lower satisfaction with social contact and recreational activities, and delayed academic life (*p* < .05). Further, the result of the multivariate logistic analysis showed that students whose diet (aOR = 0.369, 95% CI = 0.211–0.643) and schedule (aOR = 0.467, 95% CI = 0.273–0.801) were regular were less likely to be anxious. However, college students whose academic life were affected were twice as likely to be anxious as those who did not (aOR = 2.045, 95% CI = 1.210–3.454).

#### 3.3.4 Factors correlated with depression

Whether college students had depression or not was significantly correlated with diet, schedule, satisfaction with social contact and recreational activities, and academic life (*p* < .05). Students with a regular diet (aOR = 0.402, 95% CI = 0.233–0.692) presented less depression. Having a regular schedule (aOR = 0.401, 95% CI = 0.241–0.666) was possibly associated with depression. College students whose academic life was delayed (aOR = 1.964, 95% CI = 1.211–3.185) were more likely to get depressed (Table 3).

## 4 DISCUSSION

This study aims to present the prevalence of stress, anxiety, and depression of local college students in Wuhan and their correlates during the quarantine. Results showed that the population of respondents who had stress, anxiety, and depression accounted for 16.87%, 28.68%, and 35.12%, with the rate decreasing from mild to severe. The trend of prevalence distribution is consistent with what was found in Chinese college students (J. Liu, Zhu, et al., 2020), which can be explained by the mechanism of psychological symptoms under stressful life events. Previous research about refugees (von Werthern et al., 2018) and college students with adverse childhood experiences (Karatekin, 2018) found that the prevalence of stress, anxiety, and depression correlates with the time of isolation and the number of stressors. As fewer college students got a higher extent of stimulation from stressors, the prevalence of psychological symptoms decreased with severity. However, the overall prevalence of psychological symptoms of local college students in Wuhan is different from other places in China. Compared with this study, C. Wang, Pan, Wan, Tan, McIntyre, et al. (2020) previously found that only 8.1%, 28.8%, and 16.5% of the Chinese general population had the above symptoms, also, a study conducted among 746 217 college students in China found a lower prevalence of anxiety (11.0%) and depression (21.1%) (Ma et al., 2020), which suggests that the psychological state of college students living in the hardest‐hit areas are more worrying. This result is consistent with what was previously found during SARS (Lau et al., 2006), the Wenchuan and Lushan earthquakes (Xie et al., 2017), and the Ebola outbreak (Jalloh et al., 2018) that the psychological problems of the general population in the hardest‐hit areas during public health incidents are more prominent than other places.

Among 222 students with medical backgrounds who were stressed, 45 (20.27%) majored in medicine, 185 (83.33%) had families engaged in medical‐related careers, and 8 (3.6%) got both. This is inconsistent with previous research that higher health literacy could reduce medical students' negative emotions and medical college students during the pandemic were at lower risk of depression, anxiety, and insomnia (Nguyen et al., 2020). The difference can be related to the higher academic pressure on medical students (Gazzaz et al., 2018) and worries about their family members working on the frontline due to the full investment of medical resources in Wuhan.

Disrupted living rhythm during the pandemic can lead to irregular diet and rest, which were risk factors for stress, anxiety, and depression. 16.9% of the local college students reported having an irregular diet and 29.6% reported having an irregular schedule. An irregular diet includes unhealthy eating habits and food choices. Ammar et al. (2020) found that the behaviors of gluttony, snacking, and unhealthy food intake increased during the COVID‐19 pandemic, which corroborated the increasing incidence of irregular diet. The result of He Ling, et al. (2020) on the Chinese population shows that emotional eating can lead to stress, anxiety, and depression (Di et al., 2020). Moreover, a high‐fat and high‐sugar diet can lead to a process similar to “addiction” and further aggravate the symptoms (Carter et al., 2019). This study also found that it took more than 1 week for 17.64% of the respondents to stock up on enough food, difficulty in obtaining fresh food may also trigger negative emotions. An irregular schedule contains circadian rhythm disorder and decreased sleeping quality. Quarantine under the COVID‐19 pandemic can cause circadian rhythm disorder by disturbing major life events which can keep the biological clock synchronized (Morin et al., 2020). According to Goldstein & Walker (2014), lack of sleep will lead to a series of emotional disorders by influencing the regulation of emotions. Consistently, Ghrouz et al. (2019) found that poor sleeping quality is significantly correlated with anxiety and depression and it has been proven by Riemann (2018) that sleeping problems including insomnia and nightmares have a decisive negative impact on mental health. Thus, it can be inferred that the poor psychological state of college students in Wuhan is inseparable from an irregular schedule.

Additionally, although online teaching was adopted due to the pandemic, this new form of learning brought out many problems, and other related academic activities including laboratory experiments, social examinations, graduation projects, and job hunting had to be postponed to a certain extent. We found that college students whose academic arrangements were negatively affected were more likely to be stressed and anxious, which is identical to the findings of Fruehwirth et al. (2021) that distance learning is a risk factor for anxiety and depression. It is worth mentioning that the impact on the respondents differed in grades. Among all the students, the proportion of students considering pandemics as negative events to their academic progression was particularly high in the graduation grade (77.42%), which was consistent with a previous study (Zhai & Du, 2020). Notably, despite graduation students got the highest proportion of being negatively affected, the prevalence of stress, anxiety, and depression was higher in students in other grades. One possible explanation was that they had a more tightly packed curriculum and were not used to online learning (Li et al., 2020).

## 5 LIMITATIONS AND STRENGTH

The strength of this study lies in its target city, population, and time. Previous studies have proven that place of residence (hit hardest or not by pandemics) was a risk factor for negative psychological responses (Talevi et al., 2020) and students' status was significantly associated with a greater psychological impact of the outbreak and higher levels of stress, anxiety, and depression (C. Wang, Pan, Wan, Tan, Xu, et al., 2020). We provide a figure of the psychological state of college students who lived in the hardest‐hit place in China and went through the sudden quarantine at the peak time of the outbreak.

There are also several limitations to this study. The first is snowball sampling based on social networks, forwarding, and voluntary participation may introduce selection bias, resulting in an unbalanced proportion of students above the fourth grade compared with that of others. Second, this study based on self‐report tools may introduce some systematic deviations compared with the survey measures based on interviews. Third, due to the nature of the cross‐sectional study, causal relationships between the variables can not be determined.

## 6 CONCLUSION AND CLINICAL IMPACTS

This study showed that during the unexpected quarantine, different levels of stress, anxiety, and depression symptoms were prevalent among local college students in Wuhan. These acute mental disorders were found to be associated with having a medical background, having relatives or friends confirmed or suspected, having an irregular diet or schedule, and academic progression being disrupted.

To improve the psychological state of college students, measures should be taken by the departments concerned. For policymakers and science popularization workers, they should try their best to reduce the spread of rumors, let students have a more accurate understanding of COVID‐19, and at the same time, vigorously encourage students to actively carry out pandemic prevention and control work, such as getting vaccinated, insisting on wearing masks, keeping social distance and so on, so as to reduce the possible risk of sickness for themselves and their families. For school administrators, they should make efforts on coordinating students' academic life (especially graduation arrangements) and helping them achieve a better adaption to online study as soon as possible.

Because the virus will coexist with humans for a long time in the future, pandemic prevention and control will surely enter a phase of normalcy, all these notable factors found in this study and efforts should be taken into consideration for the intervention and improvement of college students' psychological state in the long term.

## CONFLICTS OF INTEREST

The authors declare no conflicts of interest.

## Data Availability

The data that support the findings of this study are available from the corresponding author upon reasonable request.

## References

[pits22699-bib-0001] Ammar, A. , Brach, M. , Trabelsi, K. , Chtourou, H. , Boukhris, O. , Masmoudi, L. , Bouaziz, B. , Bentlage, E. , How, D. , Ahmed, M. , Müller, P. , Müller, N. , Aloui, A. , Hammouda, O. , Paineiras‐Domingos, L. L. , Braakman‐Jansen, A. , Wrede, C. , Bastoni, S. , Pernambuco, C. S. , … Hoekelmann, A. (2020). Effects of COVID‐19 home confinement on eating behaviour and physical activity: Results of the ECLB‐COVID19 International Online Survey. Nutrients, 12(6):1583. 10.3390/nu12061583 PMC7352706 32481594

[pits22699-bib-0002] Antony, A. M. , Bieling, P. J. , Cox, B. J. , Enns, M. W. , & Swinson, R. P. (1998). Psychometric properties of the 42‐item and 21‐item versions of the Depression Anxiety Stress Scales in clinical groups and a community sample. Psychological Assessment, 10(2), 176–181.

[pits22699-bib-0003] Auerbach, R. P. , Mortier, P. , Bruffaerts, R. , Alonso, J. , Benjet, C. , Cuijpers, P. , Demyttenaere, K. , Ebert, D. D. , Green, J. G. , Hasking, P. , Murray, E. , Nock, M. K. , Pinder‐Amaker, S. , Sampson, N. A. , Stein, D. J. , Vilagut, G. , Zaslavsky, A. M. , & Kessler, R. C. (2018). WHO World Mental Health Surveys International College Student Project: Prevalence and distribution of mental disorders. Journal of Abnormal Psychology, 127(7), 623–638. 30211576 10.1037/abn0000362 PMC6193834

[pits22699-bib-0004] Bao, Y. , Sun, Y. , Meng, S. , Shi, J. , & Lu, L. (2020). nCoV epidemic: Address mental health care to empower society. Lancet, 395(10224), e37–e38. 32043982 10.1016/S0140-6736(20)30309-3 PMC7133594

[pits22699-bib-0038] Bian, H. , Pan, T. , & Zhao, M. (2020). Emotion of college students in the early stage of COVID‐19 epidemic. Chinese Journal of School Health, 41(05), 668–670.

[pits22699-bib-0005] Carter, J. C. , Van Wijk, M. , & Rowsell, M. (2019). Symptoms of 'food addiction' in binge eating disorder using the Yale Food Addiction Scale version 2.0. Appetite, 133, 362–369. 30508614 10.1016/j.appet.2018.11.032

[pits22699-bib-0039] Chen, X. , & Hao, W. (2020). Investigation on negative emotion of higher vocational students during the COVID‐19—Take Zhongshan polytechnic as an example. Psychologies, 15(18), 79–80.

[pits22699-bib-0006] Fruehwirth, J. C. , Biswas, S. , & Perreira, K. M. (2021). The Covid‐19 pandemic and mental health of first‐year college students: Examining the effect of Covid‐19 stressors using longitudinal data. PLOS ONE, 16(3), e0247999. 33667243 10.1371/journal.pone.0247999 PMC7935268

[pits22699-bib-0040] Gao, H. , Wang, X. , & Li, Q. (2020). Investigation and analysis of college students' psychological status at home during the COVID‐19 epidemic. Medical Diet and Health, 18(12), 213–215.

[pits22699-bib-0007] Gazzaz, Z. J. , Baig, M. , Al Alhendi, B. , Al Suliman, M. , Al Alhendi, A. S. , Al‐Grad, M. , & Qurayshah, M. (2018). Perceived stress, reasons for and sources of stress among medical students at Rabigh Medical College, King Abdulaziz University, Jeddah, Saudi Arabia. BMC Medical Education, 18(1), 29. 29471824 10.1186/s12909-018-1133-2 PMC5824476

[pits22699-bib-0008] Ghrouz, A. K. , Noohu, M. M. , Dilshad Manzar, M. , Warren Spence, D. , BaHammam, A. S. , & Pandi‐Perumal, S. R. (2019). Physical activity and sleep quality in relation to mental health among college students. Sleep & Breathing, 23(2), 627–634. 30685851 10.1007/s11325-019-01780-z

[pits22699-bib-0009] de Girolamo, G. , Dagani, J. , Purcell, R. , Cocchi, A. , & McGorry, P. D. (2012). Age of onset of mental disorders and use of mental health services: Needs, opportunities and obstacles. Epidemiology and Psychiatric Sciences, 21(1), 47–57. 22670412 10.1017/s2045796011000746

[pits22699-bib-0010] Goldstein, A. N. , & Walker, M. P. (2014). The role of sleep in emotional brain function. Annual Review of Clinical Psychology, 10, 679–708. 10.1146/annurev-clinpsy-032813-153716 PMC4286245 24499013

[pits22699-bib-0041] He, L. , Gao, Y. , Gao, X. , & Lei, X. (2020). Sleep patterns and physical and mental health of residents during the COVID‐19: Susceptibility factors and coping strategies. Journal of Southwest University (Natural Science Edition), 42(05), 11–20.

[pits22699-bib-0011] Jalloh, M. F. , Li, W. , Bunnell, R. E. , Ethier, K. A. , O'Leary, A. , Hageman, K. M. , Sengeh, P. , Jalloh, M. B. , Morgan, O. , Hersey, S. , Marston, B. J. , Dafae, F. , & Redd, J. T. (2018). Impact of Ebola experiences and risk perceptions on mental health in Sierra Leone, July 2015. BMJ Global Health, 3(2), e000471. 10.1136/bmjgh-2017-000471 PMC5873549 29607096

[pits22699-bib-0012] Karatekin, C. (2018). Adverse childhood experiences (ACEs), stress and mental health in college students. Stress Health, 34(1), 36–45. 28509376 10.1002/smi.2761

[pits22699-bib-0013] Lau, J. T. , Yang, X. , Tsui, H. Y. , Pang, E. , & Wing, Y. K. (2006). Positive mental health‐related impacts of the SARS epidemic on the general public in Hong Kong and their associations with other negative impacts. Journal of Infection, 53(2), 114–124. 16343636 10.1016/j.jinf.2005.10.019 PMC7132442

[pits22699-bib-0014] Lei, L. , Huang, X. , Zhang, S. , Yang, J. , Yang, L. , & Xu, M. (2020). Comparison of prevalence and associated factors of anxiety and depression among people affected by versus people unaffected by quarantine during the COVID‐19 epidemic in Southwestern China. Medical Science Monitor, 26, e924609. 32335579 10.12659/MSM.924609 PMC7199435

[pits22699-bib-0015] Li, H. Y. , Cao, H. , Leung, D. Y. P. , & Mak, Y. W. (2020). The psychological impacts of a COVID‐19 outbreak on college students in China: A longitudinal study. International Journal of Environmental Research and Public Health, 17(11), 3933. 10.3390/ijerph17113933 PMC7312488 32498267

[pits22699-bib-0016] Liu, J. , Zhu, Q. , Fan, W. , Makamure, J. , Zheng, C. , & Wang, J. (2020). Online mental health survey in a medical college in China during the COVID‐19 outbreak. Frontiers in Psychiatry, 11, 459. 32574242 10.3389/fpsyt.2020.00459 PMC7237734

[pits22699-bib-0017] Liu, N. , Zhang, F. , Wei, C. , Jia, Y. , Shang, Z. , Sun, L. , Wu, L. , Sun, Z. , Zhou, Y. , Wang, Y. , & Liu, W. (2020). Prevalence and predictors of PTSS during COVID‐19 outbreak in China hardest‐hit areas: Gender differences matter. Psychiatry Research, 287, 112921. 32240896 10.1016/j.psychres.2020.112921 PMC7102622

[pits22699-bib-0018] Lovibond, P. F. , & Lovibond, S. H. (1995). The structure of negative emotional states: Comparison of the Depression Anxiety Stress Scales (DASS) with the Beck Depression and Anxiety Inventories. Behaviour Research and Therapy, 33(3), 335–343. 7726811 10.1016/0005-7967(94)00075-u

[pits22699-bib-0019] Ma, Z. , Zhao, J. , Li, Y. , Chen, D. , Wang, T. , Zhang, Z. , Chen, Z. , Yu, Q. , Jiang, J. , Fan, F. , & Liu, X. (2020). Mental health problems and correlates among 746217 college students during the coronavirus disease 2019 outbreak in China. Epidemiology and Psychiatric Sciences, 29, e181. 33185174 10.1017/S2045796020000931 PMC7681173

[pits22699-bib-0020] Morin, C. M. , Carrier, J. , Bastien, C. , Godbout, R. , & Canadian Sleep and Circadian Network . (2020). Sleep and circadian rhythm in response to the COVID‐19 pandemic. Canadian Journal of Public Health=Revue Canadienne de Sante Publique, 111(5), 654–657. 32700231 10.17269/s41997-020-00382-7 PMC7375451

[pits22699-bib-0021] Nguyen, H. T. , Do, B. N. , Pham, K. M. , Kim, G. B. , Dam, H. T. B. , Nguyen, T. T. , Nguyen, T. T. P. , Nguyen, Y. H. , Sørensen, K. , Pleasant, A. , & Duong, T. V. (2020). Fear of COVID‐19 scale‐associations of its scores with health literacy and health‐related behaviors among medical students. International Journal of Environmental Research and Public Health, 17(11), 4164. 10.3390/ijerph17114164 PMC7311979 32545240

[pits22699-bib-0022] Qiu, J. , Shen, B. , Zhao, M. , Wang, Z. , Xie, B. , & Xu, Y. (2020). A nationwide survey of psychological distress among Chinese people in the COVID‐19 epidemic: implications and policy recommendations. General Psychiatry, 33(2), e100213. 32215365 10.1136/gpsych-2020-100213 PMC7061893

[pits22699-bib-0023] Di Renzo, L. , Gualtieri, P. , Cinelli, G. , Bigioni, G. , Soldati, L. , Attinà, A. , Bianco, F. F. , Caparello, G. , Camodeca, V. , Carrano, E. , Ferraro, S. , Giannattasio, S. , Leggeri, C. , Rampello, T. , Lo Presti, L. , Tarsitano, M. G. , & De Lorenzo, A. (2020). Psychological aspects and eating habits during COVID‐19 home confinement: Results of EHLC‐COVID‐19 Italian online survey. Nutrients, 12(7):2152. 10.3390/nu12072152 PMC7401000 32707724

[pits22699-bib-0024] Riemann, D. (2018). Sleep hygiene, insomnia and mental health. Journal of Sleep Research, 27(1), 3. 29336095 10.1111/jsr.12661

[pits22699-bib-0025] Talevi, D. , Socci, V. , Carai, M. , Carnaghi, G. , Faleri, S. , Trebbi, E. , di Bernardo, A. , Capelli, F. , & Pacitti, F. (2020). Mental health outcomes of the CoViD‐19 pandemic. Rivista di Psichiatria, 55(3), 137–144. 32489190 10.1708/3382.33569

[pits22699-bib-0026] Wang, C. , Pan, R. , Wan, X. , Tan, Y. , Xu, L. , Ho, C. S. , & Ho, R. C. (2020). Immediate Psychological Responses and Associated Factors during the Initial Stage of the 2019 Coronavirus Disease (COVID‐19) Epidemic among the General Population in China. International Journal of Environmental Research and Public Health, 17(5), 1729. 10.3390/ijerph17051729 PMC7084952 32155789

[pits22699-bib-0027] Wang, C. , Pan, R. , Wan, X. , Tan, Y. , Xu, L. , McIntyre, R. S. , Choo, F. N. , Tran, B. , Ho, R. , Sharma, V. K. , & Ho, C. (2020). A longitudinal study on the mental health of general population during the COVID‐19 epidemic in China. Brain, Behavior, and Immunity, 87, 40–48. 32298802 10.1016/j.bbi.2020.04.028 PMC7153528

[pits22699-bib-0028] Wang, X. , Hegde, S. , Son, C. , Keller, B. , Smith, A. , & Sasangohar, F. (2020). Investigating mental health of US college students during the COVID‐19 pandemic: Cross‐sectional survey study. Journal of Medical Internet Research, 22(9), e22817. 32897868 10.2196/22817 PMC7505693

[pits22699-bib-0029] von Werthern, M. , Robjant, K. , Chui, Z. , Schon, R. , Ottisova, L. , Mason, C. , & Katona, C. (2018). The impact of immigration detention on mental health: a systematic review. BMC Psychiatry, 18(1), 382. 30522460 10.1186/s12888-018-1945-y PMC6282296

[pits22699-bib-0030] WHO . (2020). Novel Coronavirus (2019‐nCoV) Situation Report—17. Retrieved 24th Jul 2021, from https://www.who.int/docs/default-source/coronaviruse/situation-reports/20200206-sitrep-17-ncov.pdf?sfvrsn=17f0dca_4

[pits22699-bib-0031] Xie, Z. , Xu, J. , & Wu, Z. (2017). Mental health problems among survivors in hard‐hit areas of the 5.12 Wenchuan and 4.20 Lushan earthquakes. Journal of Mental Health, 26(1), 43–49. 28084103 10.1080/09638237.2016.1276525

[pits22699-bib-0032] Zhai, Y. , & Du, X. (2020). Addressing collegiate mental health amid COVID‐19 pandemic. Psychiatry Research, 288, 113003. 32315885 10.1016/j.psychres.2020.113003 PMC7162776

